# Circular RNA: metabolism, functions and interactions with proteins

**DOI:** 10.1186/s12943-020-01286-3

**Published:** 2020-12-14

**Authors:** Wei-Yi Zhou, Ze-Rong Cai, Jia Liu, De-Shen Wang, Huai-Qiang Ju, Rui-Hua Xu

**Affiliations:** 1 State Key Laboratory of Oncology in South China, Collaborative Innovation Center for Cancer Medicine, Sun Yat-sen University Cancer Center, Guangzhou, 510060 P. R. China; 2 Research Unit of Precision Diagnosis and Treatment for Gastrointestinal Cancer, Chinese Academy of Medical Sciences, Guangzhou, 510060 P. R. China

**Keywords:** CircRNA, CircRNA-protein interaction, Mechanism, Metabolism, Function

## Abstract

Circular RNAs (CircRNAs) are single-stranded, covalently closed RNA molecules that are ubiquitous across species ranging from viruses to mammals. Important advances have been made in the biogenesis, regulation, localization, degradation and modification of circRNAs. CircRNAs exert biological functions by acting as transcriptional regulators, microRNA (miR) sponges and protein templates. Moreover, emerging evidence has revealed that a group of circRNAs can serve as protein decoys, scaffolds and recruiters. However, the existing research on circRNA-protein interactions is quite limited. Hence, in this review, we briefly summarize recent progress in the metabolism and functions of circRNAs and elaborately discuss the patterns of circRNA-protein interactions, including altering interactions between proteins, tethering or sequestering proteins, recruiting proteins to chromatin, forming circRNA-protein-mRNA ternary complexes and translocating or redistributing proteins. Many discoveries have revealed that circRNAs have unique expression signatures and play crucial roles in a variety of diseases, enabling them to potentially act as diagnostic biomarkers and therapeutic targets. This review systematically evaluates the roles and mechanisms of circRNAs, with the hope of advancing translational medicine involving circRNAs.

## Introduction

Single-stranded, covalently closed circRNAs were first reported as viroids, which are pathogens of certain plants, in 1976 [1] and were first detected in human HeLa cells by electron microscopy in 1979 [2]. Later, more studies found or synthesized circular forms of RNAs in various species, including viruses [3], prokaryotes [4], unicellular eukaryotes [4, 5] and mammals [6]. With the development of high-throughput RNA-sequencing and bioinformatic tools, scientists have found that circRNA is a general feature of the human transcriptome and is ubiquitous in many other metazoans [7–9]. More recently, an increasing number of investigations have identified multiple functions of circRNAs, including serving as protein scaffolds or miR sponges and being translated into polypeptides [7, 8, 10].

The unique structure of circRNAs provides them with a longer half-life and more resistance to RNase R than linear RNAs [11], which makes them potential candidates for diagnostic biomarkers and therapeutic targets. Plenty of studies have uncovered their distinct expression signatures and crucial biological roles in a variety of diseases, such as cancer [12–14], cardiovascular disease [15], neurological disorder [16] and autoimmune disease [17]. However, the mechanisms underlying the abnormal landscape of circRNAs and how circRNAs exert physiological or pathological roles in diseases remain poorly understood. Moreover, the majority of functional studies have shown that circRNAs act as miR sponges, which are monotonous and stereotypical. Antisense transcript of cerebellar degeneration-related protein 1 (CDR1as) represents the earliest functionally studied circRNAs and expands the competing endogenous RNA (ceRNA) crosstalk network [18–20]. Interestingly, a recent study reported that CDR1as interacts with IGF2BP3 and compromises its pro-metastatic functions [21]. Another one found that CDR1as interacts with p53 and blocks it from MDM2 [22]. This inspires us that circRNAs can be versatile and previously-studied circRNAs may also possess other abilities. Actually, one circRNA can simultaneously function as both a miR sponge and a protein template [23] (or interactor [24, 25]).

Additionally, proteins are direct effectors of almost all vital activities. CircRNA-protein interactions have remarkably rejuvenated the field of circRNAs and enlightened our insights into their biological significance. However, studies on circRNA-protein interaction are still lacking, and its mechanisms fascinate scientists. Therefore, in this article, we reviewed the important progress in the metabolism and functions of circRNAs and highlighted the modes of circRNA-protein interactions. Hopefully, this review will help to reveal intricate circRNA-related issues and to develop circRNA-targeted translational research and clinical applications.

## Metabolism of circRNA

**Fig. 1:**
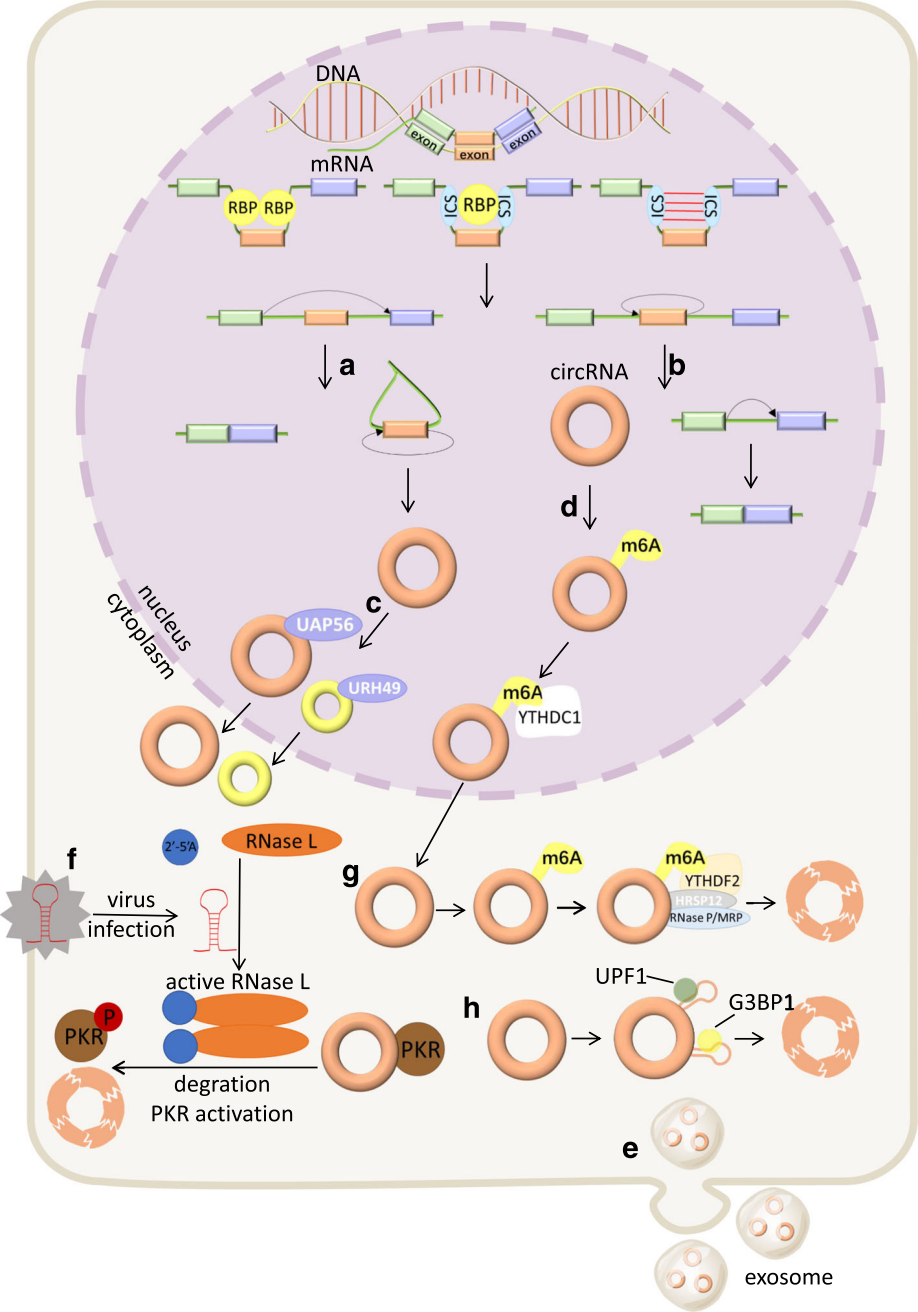
Metabolism of circRNA. *Regulation of circRNA biogenesis.* RBP can modulate circRNA biogenesis by dimerization, ICS stabilization or ICS impairment. ICS in flanking introns can facilitate exon circularization. *Biogenesis of circRNA*. **a** In the lariat model, back-spliced exons are skipped and extruded to form an intronic lariat that undergoes further back-splicing, while the remaining exons directly link with each other and form a mature mRNA. **b** In the direct model, back-splicing occurs first to form a circRNA, leaving an immature linear RNA containing introns. *Localization of circRNA* **c|** Long (> 800 nt) or short circRNAs can be translocated to the cytoplasm with the assistance of UAP56 or URH49, respectively. **d** CircRNAs can be translocated to the cytoplasm in m6A-dependent manner mediated by YTHDC1. **e** CircRNAs can be excreted to the extracellular space by exosomes. *Degradation of circRNA*. **f** Upon viral infection, RNase L activated by 2′-5′-oligoadenosine(2′-5′A) causes the global degradation of circRNAs, which relieves the suppression of PKR. **g** M6A-containing circRNAs can be recognized by YTHDF2, which interacts with the RNase P/MRP complex bridged by HRSP12, and then the complex endoribonucleolytically cleaves circRNAs. **h** UPF1 and G3BP1 can bind to imperfect base-paired regions of circRNAs and induce their degradation

In this module, we briefly introduce the cutting-edge investigations into circRNA metabolism and discuss how its modification influences metabolic programs (Fig. 1). To date, the upstream regulatory machinery of circRNA remains intriguing.

### Biogenesis

Generally, pre-mRNA transcribed by RNA polymerase II (Pol II) contains introns and exons, followed by a 7-methylguanosine cap and poly-adenosine tail adding to its 5′- and 3′-ends, respectively. Then with the assistance of spliceosomes, pre-mRNA undergoes splicing at canonical splice sites (5′-GU and 3′-AG at introns) to become mature and translatable. CircRNA is generated by a special alternative splicing manner termed back-splicing, in which the 3′-end of an exon ligates to the 5′-end of its own or an upstream exon through a 3′,5′-phosphodiester bond, forming a closed structure with a back-splicing junction site [7, 8, 26]. Initially, circRNAs were regarded as splicing errors containing so-called “scrambled exons” [27].

According to the order of splicing events and different intermediates, two models of biogenesis were proposed [28] and validated [29]: the lariat model and the direct back-splicing model [26]. Recently, an excellent study assembled the spliceosome E complex on pre-mRNA and carried out structural and biochemical analyses, proposing an integral model for intron definition, exon definition, remodeling and the back-splicing-mediated circRNA biogenesis [30].

### Regulation

CircRNA biogenesis relies on canonical splicing machinery, including splice signal sites and spliceosomes [31]. However, inhibiting the pre-mRNA processing machinery shifts the output of genes to circRNAs [32], which implies that there is competition between circRNAs and their linear counterparts. Cis-elements (intronic complementary sequences, ICSs) and trans-factors (RNA binding proteins, RBPs) can regulate circRNA production [33].

Inverted repeated Alu pairs in flanking introns facilitate exon circularization, while pairs in the same intron promote the extrusion of themselves and canonical splicing [34]. Mammalian-wide interspersed repeat family elements also contribute to the circularization of circRNAs [35]. Additionally, a group of RBPs modulate circRNA biogenesis by binding to flanking introns. Some directly draw introns into proximity and facilitate circularization [33, 36], and others stabilize [37] or impair [38] Alu pairs to promote or prevent back-splicing, respectively.

A recent study found that N6-methyladenosine (m6A) controls circRNA biogenesis. Methyltransferase-like 3 (METTL3) or YTH domain-containing 1 (YTHDC1) depletion both regulate approximately 20% of a subset of circRNAs, with no significant changes in the linear isoforms [39]. Another study found that alkB homolog 5 (ALKBH5) inhibition increases the production of translatable circRNAs through m6A enrichment at junction sites. Meanwhile, the m6A-modified start codon is recognized by YTHDF3 and mediates translation initiation [40]. However, whether there are any other regulatory factors and how m6A deposition affects the choice between back and canonical splicing remain unclear.

### Localization

CircRNAs formed from exons are generally localized to the cytoplasm [41]. The mechanisms of their nuclear export remained elusive until a recent study found that UAP56 or URH49 depletion causes long or short circRNAs, respectively, to be enriched in the nucleus, which suggests that their transportation is partially length-dependent [42]. Another study showed that the nuclear export of circNSUN2 is mediated by YTHDC1 recruitment, which provides the first evidence that m6A controls circRNA translocation [43].

Additionally, scientists have identified certain intron-containing circRNAs that are retained in the nucleus and regulate their parental gene expression [44, 45]. Some exonic circRNAs are predominantly distributed in the nucleus as well and increase the nuclear retention of proteins [46] or recruit proteins to chromatin [47]. Moreover, circRNAs can be delivered by extracellular vesicle (EV) and detected in the circulation and urine [48]. The sorting of these exosomal circRNAs seems to be regulated by associated miR levels in producer cells, while the specific biological activities transferred to recipient cells are largely unknown in diverse settings [48, 49]. The packaging, delivery and absorption of them also remain elusive so far. Recently, a handful of studies identified mitochondria-located circRNAs and examined their functions, which broaden our knowledge of circRNA derivation and mitochondrial transcriptome [50–52]. Nevertheless, circRNAs located in other organelles or subcellular compartments deserve further investigations.

### Degradation

CircRNAs are stable and accumulate in many cell types, especially in neural tissues [53]. In contrast, another study reported a global reduction in circRNA abundance in highly proliferative tissues, possibly due to dilution by proliferation [54]. Regarding how circRNAs maintain a dynamic balance, recent research has shed light on the mechanisms of circRNA degradation. One study revealed that circRNAs can be globally degraded by RNase L. Endogenous circRNAs tend to form imperfect duplexes and inhibit PKR (dsRNA-activated protein kinase), while their reduction leads to aberrant PKR activation and autoimmunity [55]. Another study found that m6A-containing circRNAs are recognized by YTHDF2 that interacts with RNase P/MRP (mitochondrial RNA processing) bridged by heat-responsive protein 12 (HRSP12). Then, these circRNAs are endoribonucleolytically cleaved [56]. A more recent study proposed a structure-dependent mechanism mediated by UPF1 RNA helicase and ATPase (UPF1) and G3BP stress granule assembly factor 1 (G3BP1), which bind to highly structured base-paired regions and direct both mRNA and circRNA decay [57].

Additionally, miR-671 directs the cleavage of CDR1as in an Argonaute2 (Ago2)-dependent manner [58]. GW182 (a key component of P-body and RNAi machinery) is also involved in circRNA degradation [59]. Apart from intracellular pathways, circRNA excretion may contribute to their clearance [60]. Further studies are needed to fully understand circRNA decay mechanisms and interpret their homeostasis and differential distribution across cell types.

### Modification

M6A is the most common RNA modification and is regulated by readers, writers and erasers [61]. Emerging studies have revealed a transcriptome-wide and cell-type-specific map of m6A-containing circRNAs [62] and m6A involvement in circRNA metabolism [63]. In addition to abovementioned biogenesis [39, 40], nuclear export [43] and degradation [56], m6A also modulates circRNA translation. One study reported that m6A drives the translation initiation of circRNAs mediated by the interaction between eIF4G2 and YTHDF3, which is enhanced by METTL3/14 and suppressed by FTO [64]. Additionally, the YTHDF2 binding of m6A-markedself-circRNAs abrogates innate circRNA immunity [65]. Other types of modifications in circRNAs await further identifications.

## Functions of circRNA

**Fig. 2:**
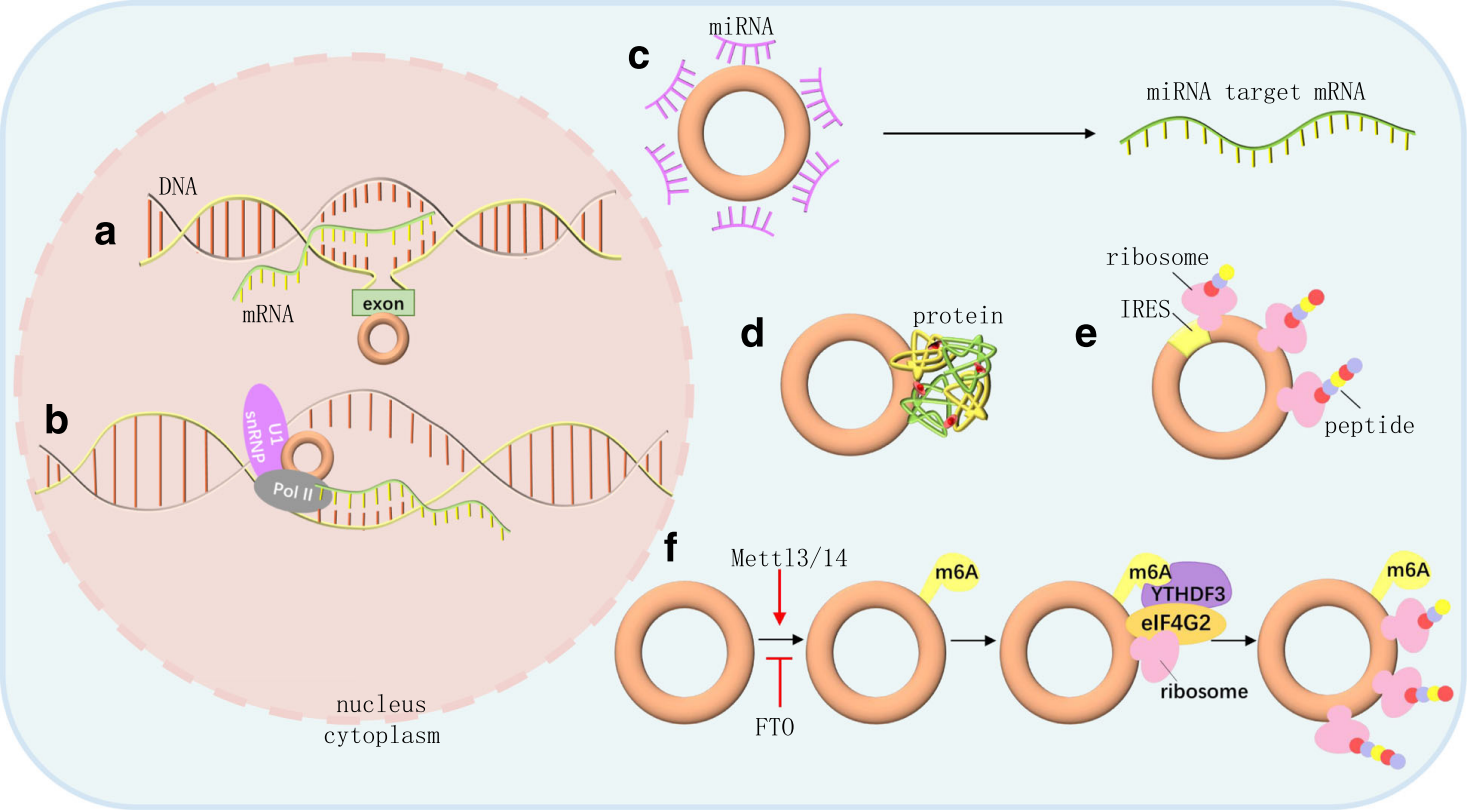
Functions of circRNA. **a** CircRNAs can bind to the host genes at their synthesis locus and cause transcriptional pausing or termination through the formation of RNA-DNA hybrid (R-loop structure), upregulating the exon-skipped or truncated transcripts. **b** EIciRNAs can combine with U1 snRNP and then interact with Pol II to enhance parental gene expression. **c** CircRNAs can act as miR sponges and upregulate the miR target mRNAs. **d** CircRNAs can interact with proteins. **e** IRES-containing circRNAs can directly recruit ribosomes and be translated. **f** M6A-containing circRNAs can be recognized by YTHDF3, which recruits eIF4G2, thus triggering translation. This process can be enhanced by METTL3/14 and suppressed by fat mass and obesity-associated protein (FTO)

In this module, we discuss how circRNAs function at the molecular level and underlying mechanisms mainly involve interactions with other molecules. CircRNAs have long been considered “non-coding” RNAs (ncRNAs) with regulatory potency [20, 66]. Later, scientists identified translatable circRNAs [67, 68], and more recent studies presented evidence for their prevalence [69, 70] (Fig. 2). Briefly, downstream pathways of circRNA are mostly related to miR sponges and other brilliant talents that circRNA yields require brand new pursuits. We will systematically review the current progress in circRNA-protein interactions and discuss this topic in detail in the next module.

### Transcriptional regulation

CircSEP3 originating from exon 6 of SEPALLATA3 increases the abundance of cognate exon 6-skipped variant by binding to the host DNA locus and forming an RNA-DNA hybrid or R-loop, resulting in transcriptional pausing and splicing factor recruitment [71]. Similarly, circSMARCA5 causes transcriptional termination at exon 15 of SMARCA5 through R-loop formation, upregulating the truncated nonfunctional isoform [72]. CircRNAs with introns retained between exons (exon-intron circRNAs, EIciRNAs) can combine with U1 small nuclear ribonucleoprotein through RNA-RNA interactions between snRNA and EIciRNAs and then interact with Pol II at parental gene promoters, enhancing their expression [44]. Likewise, circular intronic RNAs (ciRNAs) formed from lariats that escape debranching can accumulate at their synthesis sites and increase parental gene expression by modulating elongating Pol II activity [45].

### MicroRNA sponging

Plenty of studies have confirmed that circRNAs exert biological functions by acting as ceRNAs or miR sponges [73, 74]. CDR1as contains 63 conserved miR-7 binding sites [20] and increases the levels of miR-7 target mRNAs, which are mostly associated with tumor progression [75, 76]. By sponging miR-7, CDR1as regulates the development of zebrafish midbrain [20] and the processes of many other diseases [77]. Apart from miRs, circPan3 binds to and stabilizes the mRNA that encodes interleukin-13 (IL-13) receptor subunit IL-13Rα1, eventually leading to the maintenance of intestinal stem cells [78]. However, the stoichiometric relationship between circRNAs and other molecules (miRs and proteins) has received more attention [7, 13]. In view of the generally low abundance of circRNAs and the limited number of binding sites, exactly how circRNAs exert sufficient/detectable effects remains unclear.

### Translation into proteins

The translation is performed by ribosomes and involves initiation, elongation, termination and ribosome recycling [79]. The Initiation on eukaryotic mRNAs involves scanning by 43S preinitiation complexes from the 5′ cap-proximal point of attachment to the initiation codon, followed by ribosomal subunit joining and factor displacement [80]. Lacking the 5′-cap and 3′-tail, circRNA can only adopt cap-independent manners. In addition to previously described m6A-mediated translation [39, 64, 81], artificial [67] and endogenous circRNAs containing an internal ribosome entry site (IRES) that directly recruits ribosomes [82], can also be translated [63]. These two approaches may be coupled with each other. For example, m6A improves the efficiency of IRES-mediated translation of circZNF609 [39, 83]. Additionally, circRNA with an infinite ORF undergoes rolling circle amplification in an IRES-independent manner, leading to a hundred-fold higher productivity than linear transcript [84].

Peptides encoded by circRNAs are generally truncated and their functions are mostly analogous to the full-length protein counterparts (circFBXW7-185aa [23, 85]). However, some proteins originating from circRNAs exert functions independent of or even opposed to those of their host gene products (circFNDC3B-218aa [86]). These results broaden the range of human proteome. However, the regulatory mechanisms of circRNA translation and the processes of elongation and termination are still not completely understood.

## CircRNA-protein interactions

**Fig. 3:**
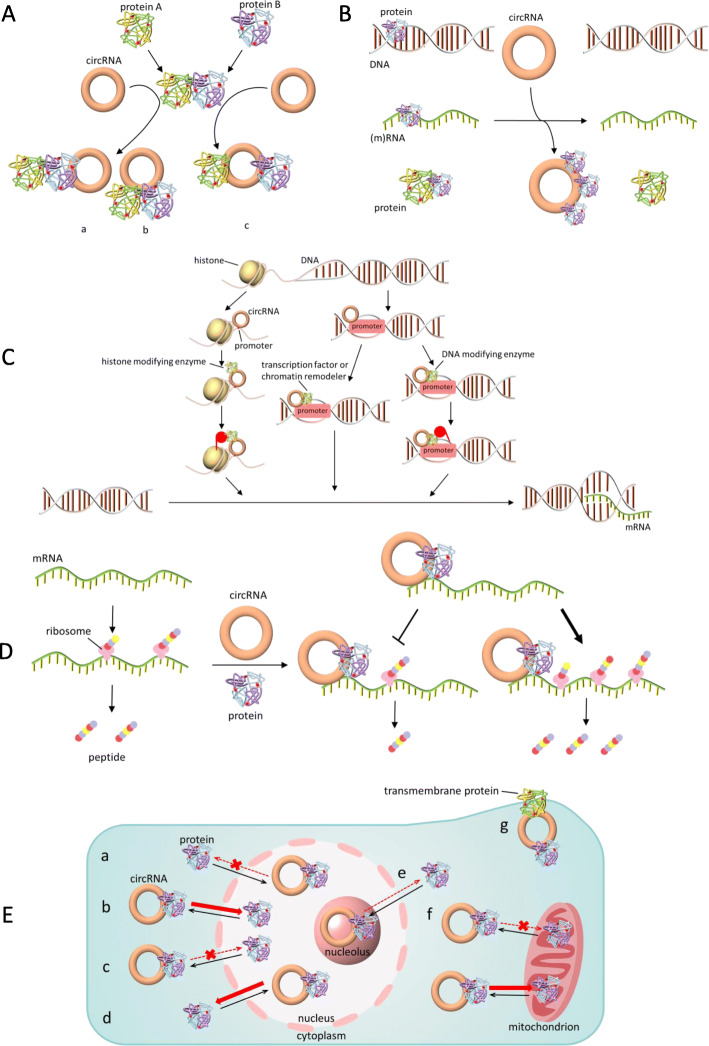
CircRNA-protein interactions. **A** (a) CircRNA binds to both proteins and strengthens their interaction. (b) CircRNA binds to protein A and reinforces its interaction with protein B, which does not directly bind to circRNA. (c) CircRNA binds to both proteins that originally combine with each other and then disrupts their interaction. **B** CircRNA blocks proteins from interacting with DNA, RNA or other proteins, thus compromising their original functions. **C** CircRNA recruits transcription factors, chromatin remodelers and DNA or histone modifying enzymes to the promoters and alters transcription (including activating and inhibiting). **D** CircRNA helps RBPs to combine with mRNA and stabilizes mRNA (indirectly promoting translation) or directly regulates translation (including promoting and inhibiting). **E** (**a**) Nuclear circRNA causes the nuclear retention of proteins. (**b**) Cytoplasmic or shuttling circRNA facilitates the nuclear import of proteins. (**c**) Cytoplasmic circRNA causes the cytoplasmic retention of proteins. (**d**) Nuclear or shuttling circRNA facilitates the nuclear export of proteins. (**e-g**) Furthermore, circRNAs can transport proteins to the nucleolus, mitochondria and membrane, respectively

A handful of studies have reported that circRNAs function by interacting with proteins [87–89]. In addition to the abovementioned RBPs that participate in circRNA metabolism [33, 36], and circRNAs that interact with Pol II [44, 45], and miR-sponging circRNAs associated with Ago2 [58], a group of circRNAs serve as protein decoys, scaffolds and recruiters in diverse physiological and pathological contexts. One circRNA may exclusively bind to a single protein or interact with multiple proteins under different circumstances. One RBP can also combine with a subgroup of circRNAs and form circRNA-protein complex (circRNP) families (IGF2BP3) [90]. However, bioinformatic analyses indicated that circRNAs possess a lower RBP binding density than linear RNAs [91], which implies that many circRNAs are incapable of interacting with proteins. Thus, urgent solutions are needed to more efficiently carry out studies on circRNA-protein interactions. In this module, we classify circRNA-protein interactions into five sections (Fig. 3). Proteins involved in circRNA upstream processes (metabolism), and some special species (e.g., Ago2 and Pol II) will not be included (all relevant studies are listed in Table 1).

**Table 1:** Known circRNA-protein interactions classified by manners of action

Manner	CircRNA	Protein	Direct Effect	Biological Function	Correlation^**a**^	Refs
**Altering interactionsbetween proteins**	circFoxo3	p53, MDM2	induce mutant p53 ubiquitination	pro-apoptosis	P	[92]
	circFoxo3	p21, CDK2	dampen the activity and accessibility of CDK2	cell cycle arrest in G1 phase	P	[93]
	circNfix	YBX1, Nedd4l	induce YBX1 ubiquitination and inhibit its nucleartranslocation	cell cycle arrest, anti-angiogenesis,anti-regeneration	U	[25]
	circADD3	CDK1, EZH2	phosphorylate EZH2 and induce its ubiquitination	anti-metastasis	P	[94]
	circAmotl1	PDK1, AKT	phosphorylate AKT and promote pAKT nucleartranslocation	anti-apoptosis, cardio-protection	P	[95]
	circGLI1	p70S6K2, GSK3β	phosphorylate GSK3β	pro-metastasis	P	[96]
	circCTNNB1	DDX3, YY1	transactivate YY1	tumor progression	P	[97]
	circCUX1	EWSR1, MAZ	transactivate MAZ	pro-Warburg effect, tumor progression	P	[98]
	circACC1	β and γ subunits of AMPK	stabilize and activate AMPK	metabolic reprogramming	N	[99]
	circCcnb1	H2AX, (wild-type p53)	free Bclaf1 from p53	cell survival	P	[100]
	circCcnb1	H2AX, (Bclaf1)	wrap Bclaf1 by H2AX	cell death	N	[100]
	circCcnb1	Ccnb1, CDK1	deactivate Ccnb1 and retain it in the cytoplasm	cell cycle arrest in G2 phase	N	[101]
**Blocking proteinsfrom DNA**	circHuR	CNBP	sequester CNBP from the HuR promoter	tumor suppression	N	[102]
	circSCMH1	MeCP2	tether MeCP2 and relieve its repression upon thetarget gene transcription	neuroprotection post stroke	P	[103]
	circSamd4	PURA, PURB	tether PURA/B and relieve their repression uponMHC transcription	pro-myogenesis	U	[104]
	ACR	DNMT3B	tether DNMT3B and decrease the methylation of thePink1 promoter	anti-autophagy	U	[105]
	cia-cGAS	cGAS	block cGAS from self-DNA and inhibit its enzymatic activity	maintaining HSCs quiescent	U	[106]
**Blocking proteinsfrom RNA**	circSMARCA5	SRSF1	tether SRSF1 and suppress its splicing activity (SRSF3, PTBP1)	anti-migration	N	[107]
	circSMARCA5	SRSF1	tether SRSF1 and suppress its splicing activity (VEGFA)	anti-angiogenesis	N	[108]
	circPABPN1	HuR	sequester HuR and destabilize PABPN1 mRNA	anti-proliferation	N	[109]
	circPABPN1	HuR	sequester HuR and destabilize Atg16L1 mRNA	anti-autophagy	U	[110]
	circZKSCAN1	FMRP	sequester FMRP from combing with CCAR1 mRNA	anti-stemness, tumor quiescence	U	[111]
	circMTO1	TRAF4	deactivate Eg5 translation	chemosensitization	U	[112]
	circMMP9	AUF1	relieve the inhibition of MMP9 mRNA	pro-metastasis	P	[113]
	circANRIL	PES1	prevent pre-rRNA maturation and impair ribosome biogenesis	anti-atherosclerosis	N	[114]
	circPPM1F	HuR	sequester HuR and destabilize PPM1F mRNA	M1 macrophage activation	N	[115]
**Blocking proteinsfrom proteins**	circGSK3β	GSK3β	block GSK3β from β-catenin	pro-metastasis	N	[116]
	CDR1as	p53	block p53 from MDM2	tumor suppression	U	[22]
	circ102171	CTNNBIP1	block CTNNBIP1 from β-catenin	tumor progression	U	[117]
	circH19	PTBP1	tether PTBP1 and inhibit its ability to cleave and activate SREBP1	adipogenesis	U	[118]
	circECE1	c-myc	block c-myc from SPOP	pro-Warburg effect	P	[119]
	SCAR	ATP5B	block mPTP from CypD	anti-metaflammation	U	[50]
**Recruiting transcriptionfactors to chromatin**	circRHOT1	TIP60	recruit TIP60 to the NR2F6 promoter and initiate transcription	tumor progression	P	[47]
	circAnks1a	YBX1	recruit YBX1 to the VEGFB promoter and activate transcription	central sensitization, painbehavioral hypersensitivity	U	[120]
	circ0005276	FUS	recruit FUS to the XIAP promoter	tumor progression	P	[121]
	circPOK	ILF2/3 complex	potentiate the affinity of ILF2/3 to the IL-6 promoter	pro-angiogenesis	N	[122]
**Recruiting modifyingenzymes to chromatin**	circFECR1	TET1	induce demethylation and activate FLI1 transcription	pro-tumorigenesis	P	[123]
	circMRPS35	KAT7	induce the acetylation of H4K5 and activate FOXO1/3a transcription	tumor suppression	U	[124]
	circAGFG1	EZH2	induce H3K27me3 of the p53 promoter	tumor progression	U	[125]
	circLRP6	LSD1, EZH2	induce H3K27me3 and H3K4me2 of the KLF2 and APC promoter	tumor progression	P	[126]
**Recruiting chromatinremodelers**	circDONSON	NURF complex	recruit the NURF complex to the SOX4 promoter and activate itstranscription	tumor progression	U	[127]
	circKcnt2	NuRD complex	recruit the NuRD complex to the Batf promoter and inhibit itstranscription	anti-inflammation	U	[128]
**Ternary complexesregulating RNA stability**	circNSUN2	IGF2BP2, (HMGA2 mRNA)	stabilize HMGA2 mRNA	pro-metastasis	P	[43]
	circPOK	ILF2/3, (mRNA of IL-6 andVEGF)	stabilize the mRNA of IL-6 and VEGF	tumor progression, pro-angiogenesis	N	[122]
	circFNDC3B	IGF2BP3, (CD44 mRNA)	stabilize CD44 mRNA	tumor progression	P	[129]
**Ternary complexesregulating translation**	circMALAT1	Ribosome, (PAX5 mRNA)	retard PAX5 translation	self-renewal of HCC stem cells	U	[130]
	circYap	eIF4G, PABP, (Yap mRNA)	interrupt the assembly of Yap translation initiation machinery	tumor suppression	N	[131]
	circMYBL2	PTBP1, (FLT3 mRNA)	promote FLT3 translation	AML progression	P	[132]
**Translocating proteinsto the nucleus**	circAmotl1	c-myc	retain c-myc in the nucleus and increase its affinity to targets	pro-tumorigenesis	P	[46]
	circAmotl1	STAT3	facilitate the nuclear translocation of STAT3	pro-wound repair	P	[133]
	circDNMT1	p53, AUF1	facilitate the nuclear translocation of p53 and AUF1, elevatingLC3B level and stabilizing DNMT1 mRNA, respectively	pro-autophagy, anti-senescence,tumor progression	P	[134]
	circABCC1	β-catenin	redistribute β-catenin	tumor progression	U	[135]
	circSOX4	β-catenin	translocate β-catenin to the nucleus	tumor progression	P	[136]
**Translocating proteinsto the cytoplasm**	circFoxo3	ID-1, E2F1, FAK, HIF1α	retain these proteins in the cytoplasm and arrest their functions	pro-senescence	P	[137]
	circFOXP1	PTBP1	translocate PTBP1 to the cytoplasm and stabilize PKLR mRNA	pro-Warburg effect, tumor progression	N	[138]
	circSTAG1	ALKBH5	retain ALKBH5 in the cytoplasm	anti-depression	U	[139]
	circBACH1	HuR	translocate HuR to the cytoplasm	cell cycle progression	P	[140]
	circZFP609	HIF1α	retain HIF1α in the cytoplasm	anti-angiogenesis	U	[141]
	circCCAC1	EZH2	retain EZH2 in the cytoplasm	pro-metastasis	P	[24]
**Other regions**	circERBB2	PA2G4	translocate PA2G4 to the nucleolus and promote rDNA transcription	tumor progression	P	[142]
	circSKA3	Tks5, Integrin β1	recruit Tks5 to the membrane and co-localize with integrin β1;induce the formation of invadopodia	pro-invasion, pro-metastasis	P	[143]
	mecciND1, mecciND5	RPA32, hnRNPA1, TOM40	interact with TOM40 and facilitate the mitochondrial importationof RPA32 and hnRNPA1, respectively	metabolism of mtDNA and mtRNA;not clear	U	[143]
	mecciRNAs	PNPASE	control the mitochondrial importation of mecciRNAs	not clear	U	[52]
**Unknown orunconfirmedmanners**	circ0011460	PGT	just verify the interaction by RIP; increase PGT level	positive correlation with pre-eclampsia	P	[144]
	circ0075932	PUM2	just verify the interaction by RNA pull-down; increase PUM2 level	pro-inflammation, pro-apoptosis	U	[145]
	circFndc3b	FUS	just verify the interaction by RIP; decrease FUS level	pro-angiogenesis, anti-apoptosis	P	[146]
	circZNF292	LDHA	just verify the interaction by RIP; increase LDHA level	pro-glycolysis	N	[147]
	circAmotl1	pAKT	just verify the interaction by RIP; activate AKT pathway	chemoresistance	P	[148]
	circ0075804 (circE2F3)	HNRNPK	stabilize E2F3 mRNA; the formation of ternary complex is notconfirmed	pro-proliferation, anti-apoptosis	P	[149]
	circPTK2	vimentin	just verify the interaction by RNA pull-down	tumor progression	U	[150]
	circ406961	ILF2	just verify the interaction by RNA pull-down; decrease ILF2 leveland suppress the STAT3/JNK pathway	anti-inflammation (induced by PM_2.5_)	U	[151]
	circBbs9	Ccnd2	just verify the interaction by RIP; increase Ccnd2 level	pro-proliferation	U	[152]
	circHECTD1	ZC3H12A	reduce ZC3H12A ubiquitination by attenuatinginteraction between ZC3H12A and HECTD1; this study only showscircHECTD1 negatively correlates with HECTD1	deactivation and pro-apoptosis ofalveolar macrophage activated bysilica	N	[153]
	circFOXK2	YBX1, hnRNPK	enhance the interaction of YBX1 and hnRNPK with NUF2 andPDXK; lack a detailed mechanism	tumor progression	N	[154]
	circMUC16	ATG13	just verify the interaction by RNA pull-down; increase ATG13 level	pro-autophagy	P	[155]
	circUBR5	QKI, NOVA1 (U1 snRNA)	probably participate in RNA splicing; lack a detailed mechanism	non-functional phenotype	U	[156]
	circNF1–419	Dynamin-1, Adaptor protein2 B1 (AP2B1)	just verify the interaction by RNA pull-down and RIP; lack a detailed mechanism	pro-autophagy, senile dementiadelay	U	[157]
	circNOL10	SCML1	just verify the interaction by RNA pull-down; increase SCML1 level	tumor suppression	U	[158]
	circHipk3	Notch1 intracellular domain(N1ICD)	verify the interaction by RNA pull-down and RIP; increase N1ICDexpression, stability, acetylation; lack a detailed mechanism	cardiac regeneration	U	[159]
	cIARS	ALKBH5	inhibit the ALKBH5-mediated interaction between Beclin1 andBcl-2; lack a detailed mechanism	pro-autophagy, pro-ferroptosis	U	[160]

^a^approximate functional correlation between circRNAs and their parental genes. *P* positive, *N* negative, *U* unknown or unrelated

### Altering interactions between proteins

In this section, we summarize three modes of circRNA-protein interactions: (1) one circRNA binds to both proteins and cements their interaction; (2) one circRNA binds to protein A and cements or dissociates its interaction with protein B that does not directly bind to circRNA; and (3) one circRNA binds to both proteins, which originally combine with each other, dissociating their interaction. The circRNA-protein A/B ternary (or more) complex arises in all three modes, but the effects are different.

#### *Cementing interactions between proteins*

In the first mode, circRNA mainly mediates posttranslational modifications (ubiquitination and phosphorylation) of protein A catalyzed by protein B or the transactivation of protein A by protein B, followed by a subsequent downstream cascade. Silencing circFoxo3 reinforces cell viability, while its overexpression enhances tumor sensitivity to chemotherapy by potentiating apoptosis. Mechanistically, circFoxo3 interacts with p53 and mouse double minute 2 (MDM2), enhancing p53 ubiquitination. This also occupies MDM2 and relieves Foxo3 ubiquitination [92]. CircFoxo3 also increases Foxo3 translation by sponging miRs [161]. In addition, circFoxo3 is associated with cell cycle. The circFoxo3-p21-CDK2 ternary complex reinforces the interaction of CDK2 with p21 (CDK inhibitor 1A) and dampens CDK2 phosphorylation activity. This impedes the formation of CDK2/cyclin E and CDK2/cyclin A complexes, and thus blocks G1/S transition and S progression, respectively, ultimately leading to cell cycle arrest in G1 phase [93]. This study addressed that a circRNA modulates protein-protein interactions for the first time.

Similarly, the loss of circNfix promotes cardiomyocyte proliferation and angiogenesis, and inhibits apoptosis post myocardial infarction. Mechanistically, circNfix reinforces the interaction between Y-box binding protein 1 (YBX1) and NEDD4-like E3 ubiquitin ligase (Nedd4l), inducing YBX1 ubiquitination. CircNfix also inhibits the nuclear translocation of YBX1, which binds to the promoters of cyclin A2 and cyclin B1 and activates transcription. In addition, circNfix sponges miR-214 and increases glycogen synthase kinase 3 beta (GSK3β) expression, subsequently repressing VEGF secretion and β-catenin activity [25]. Other examples of this mode, such as circADD3 [94], circAmotl1 [95], and circGLI1 [96] are listed in Table 1.

Regarding transactivation, circCTNNB1 promotes cancer progression by enhancing the transactivation of Yin Yang 1 (YY1) by DDX3 and thus upregulating target genes involved with β-catenin activation [97]. This mode is also illustrated by circCUX1, which promotes aerobic glycolysis and neuroblastoma progression by strengthening the transactivation of MYC-associated zinc finger protein (MAZ) by EWS RNA binding protein 1 (EWSR1) [98].

An outstanding study revealed that in metabolic adaptation to serum deprivation, the production of acetyl-CoA carboxylase 1 (ACC1) RNA switches from the linear to the circular form circACC1, which facilitates the assembly of the AMP-activated protein kinase (AMPK) holoenzyme by combining with the regulatory β and γ subunits and enhancing their interaction. This also stabilizes and activates AMPK, which inhibits anabolism and boosts catabolism, thus promoting β-oxidation and glycolysis. A tetramer is formed by AMPK and circACC1; however, circACC1 does not directly combine with the α subunit or enhance its interaction with another two subunits. The mechanism through which ACC1 pre-mRNA shifts to back-splicing under metabolic stress remains unexplored [99].

The second mode can be exemplified by circCcnb1. In p53 wild-type cells, circCcnb1 precipitates p53 bridged by H2A.X variant histone (H2AX), while in p53 mutant cells, it precipitates Bclaf1 also bridged by H2AX. However, circCcnb1 cannot directly access p53 or Bclaf1. The wild-type p53 has a greater affinity to H2AX than Bclaf1 and circCcnb1 augments this interaction, and thus the former ternary complex allows Bclaf1 to bind to Bcl2 and results in cell survival. While the mutant p53 is unable to bind to H2AX, and thus the latter complex wraps Bclaf1 by H2AX and results in cell death [100].

#### *Dissociating interactions between proteins*

In addition to H2AX, circCcnb1 can simultaneously interact with Ccnb1 and CDK1 and dissociates their interaction by forming a large ternary complex. This also decreases their nuclear translocation, thus arresting Ccnb1 function and decelerating cell cycle [101]. The situation in which a circRNA binds to protein A and dissociates its interaction with protein B that does not directly bind to circRNA is sorted into the third type (“Blocking proteins from other proteins”) of the next section. Actually, this disruption of interactions is the same as blocking protein A from B.

Therefore, we see the diversity of relationships between circRNA and two proteins and foresee the complications that arise when three or more proteins are involved. However, apart from mutual binding sites or sequences, little is known about exactly how circRNA alters protein-protein interactions. We speculate that circRNAs probably change the spatial distance of proteins, expose or cover their activity sites, and allosterically transform their conformation fit for interactions. More investigations and novel technologies are warranted to validate these hypotheses.

### Tethering or sequestering proteins

In this section, circRNA combines with only one protein and compromises original function or creates new effects. We classify relevant studies into three types based on the downstream consequences: circRNA blocks proteins from interacting with DNA, RNA or other proteins.

#### *Blocking proteins from DNA*

In the first type, DNA binding proteins (e.g., transcription factors, TFs) represent the majority, and thus circRNA adversely alters transcription. CircHuR inhibits gastric cancer (GC) proliferation, invasion and metastasis by sequestering CCHC-type zinc finger nucleic acid binding protein (CNBP) from the human antigen R (HuR) promoter, thus downregulating HuR and repressing tumor [102]. Similarly, circSCMH1 delivered by EVs improves neuronal plasticity, and inhibits glial activation and immune cell infiltration post-stroke by sequestering methyl-CpG binding protein 2 (MeCP2) and compromising its role in transcriptional repression [103]. CircSamd4 [104] and autophagy-related circRNA (ACR) [105] also conform to this type.

Cyclic GMP-AMP (cGAMP) synthase (cGAS) is a DNA sensor that catalyzes cGAMP synthesis when it binds to DNA. Then, cGAMP activates the STING pathway and turn on type I interferon expression. This evolves as a defense mechanism against microbial infections, while cGAS activated by self-DNA triggers autoimmunity [162]. Under homeostatic conditions, circRNA antagonist for cGAS (cia-cGAS) occupies cGAS and avoids its binding with self-DNA. Meanwhile, this interaction inactivates synthase activity, thereby abrogating the cGAS-mediated production of type I interferon and protecting long-term hematopoietic stem cells (LT-HSCs) from exhaustion [106].

#### *Blocking proteins from RNA*

The second type introduces a group of RBPs that control mRNA splicing, stability and translation. Therefore, circRNA indirectly intervenes in posttranscriptional processes. CircSMARCA5 inhibits glioblastoma multiforme (GBM) cell migration by sequestering serine and arginine rich splicing factor 1 (SRSF1) that enhances exon 4 skipping in SRSF3 pre-mRNA. Both SRSF1 and SRSF3 (the isoform without exon 4) upregulate PTBP1, which contributes to glioma cell migration and adhesion [107, 163]. VEGFA pre-mRNA can also be alternatively spliced by SRSF1, and its aberrant splicing leads to an alteration in the ratio of the pro−/anti-angiogenic isoforms in GBM [108].

In addition to disrupting the splicing factor network, circPABPN1 sequesters HuR that stabilizes poly-adenosine binding protein nuclear 1 (PABPN1) mRNA, consequently lowering PABPN1 levels and inhibiting HeLa cell proliferation [109]. It also prevents HuR from binding to autophagy-related 16 like 1 mRNA and causes subsequent autophagy defects, triggering inflammatory bowel diseases [110]. Similar examples in which circRNAs indirectly regulate mRNA stability or translation include circZKSCAN1 [111], circMTO1 [112], circMMP9 [113] and circPPM1F [115].

CircANRIL appears to play an atheroprotective role against its linear counterpart linANRIL. The nucleolar protein pescadillo ribosomal biogenesis factor 1 (PES1) strongly interacts with circANRIL. By occupying the C-terminal lysine-rich domain of PES1, circANRIL prevents exonuclease-mediated pre-rRNA maturation and impairs ribosome biogenesis and assembly, leading to nucleolar stress. As a result, p53 is activated and accumulates in the nucleus, thus inducing apoptosis in atherosclerotic plaques [114].

#### *Blocking proteins from other proteins*

The third type has similarities to “Dissociating interactions between proteins” in the last section. In both of them, protein contact is broken. For example, circGSK3β promotes ESCC migration and invasion by decreasing β-catenin phosphorylation by GSK3β and its sequential ubiquitination [116]. CDR1as can block p53 from MDM2, thus relieving p53 ubiquitination and protecting cells from DNA damage [22]. Similar instances include circ102171 [117], circH19 [118] and circECE1 [119].

Recently, a breakthrough concerning mitochondrial genome-encoded circRNAs reported that SCAR (steatohepatitis-associated circRNA ATP5B regulator) can bind to ATP synthase subunit b (ATP5B) that resides in the mitochondrial permeability transition pore (mPTP) complex, and block mPTP from cyclophilin D, which shuts down mPTP and inhibits mitochondrial ROS output and fibroblast activation. However, endoplasmic reticulum stress induced by lipid overload (mimicking liver fibroblasts isolated from nonalcoholic steatohepatitis patients) represses PGC-1α mediated by C/EBP homologous protein (CHOP), then downregulating SCAR [50].

However, the majority of current studies have merely observed the effects of circRNA sequestering proteins and have barely explored how circRNA influences proteins. Little is known about what happens to both molecules in this pattern (conformation, stability, abundance, distribution, modification).

### Recruiting proteins to chromatin

In this section, several studies displayed novel patterns in which circRNAs bind to cis-elements and control TFs or modulate the epigenome, altering gene expression. We categorized them into three types: TFs, DNA or histone modifying enzymes and chromatin remodelers.

#### *Recruiting TFs*

Four examples showed that circRNAs recruit transcription activators to promoter regions. CircRHOT1 knockout suppresses hepatocellular carcinoma (HCC) proliferation, migration and invasion. It recruits Tat interactive protein 60 kDa (TIP60) to the nuclear receptor subfamily 2 group F member 6 (NR2F6) promoter to initiate transcription [47]. Additionally, circAnks1a is upregulated in dorsal horn neurons following spinal nerve ligation and increases central sensitization and behavioral hypersensitivity. Mechanistically, cytoplasmic circAnks1a augments the transportin-1-mediated nuclear translocation of YBX1. Then, nuclear circAnks1a recruits YBX1 to the VEGFB promoter and activates transcription. Cytoplasmic circAnks1a also sponges miR-324-3p that targets VEGFB mRNA. Elevated VEGFB contributes to neuron excitability and pain behavior caused by nerve injury [120]. Circ0005276 [121] and circPOK [122] are also in this category.

#### *Recruiting modifying enzymes*

In regard to the epigenome, covalent modifying enzymes are recruited to DNA (methylation) or histones (methylation and acetylation) to alter chromatin accessibility and turn gene expression on or off. CircFECR1 enhances breast cancer (BC) invasion by recruiting ten–eleven translocation 1 (TET1) to the promoter of its parental gene Friend leukemia virus integration 1 (FLI1), inducing DNA demethylation and activating transcription [123]. Additionally, circMRPS35 suppresses GC proliferation and invasion by recruiting lysine acetyltransferase 7 (KAT7) to the FOXO1/3a promoter, which elicits H4K5 acetylation [124]. In contrast, circAGFG1 [125] and circLRP6 [126] both recruit enhancer of zeste 2 polycomb repressive complex 2 subunit (EZH2) to target gene promoters, inducing methylation and deactivating transcription.

#### *Recruiting chromatin remodelers*

CircDONSON promotes GC proliferation, migration and invasion by recruiting nucleosome remodeling factor (NURF, a chromatin remodeler complex) to the SRY-box transcription factor 4 (SOX4) promoter to activate its transcription [127]. In contrast, circKcnt2 inhibits the innate colitis by recruiting the nucleosome remodeling deacetylase (NuRD) complex to the Batf promoter to inhibit its transcription [128].

How cells acquire new identities or undergo reprogramming in response to various environmental cues has attracted attention. The interplay between TFs and three-dimensional genome architecture triggers cell-fate decisions [164]. It will be of interest to determine how circRNAs shape the chromatin landscape and to examine the relationship between circRNAs and the transcriptome.

### Forming circRNA-protein-mRNA ternary complexes

Ternary complexes are quite common in circRNA-protein interactions, and we mentioned circRNA-protein A/B and circRNA-protein-chromatin complexes in the first and third sections, respectively. In this section, we focus on circRNA-protein-mRNA ternary complexes, which regulate mRNA stability or directly modulate translation. It is noteworthy that in circRNA-protein-nucleic acid (DNA or RNA) ternary complexes, three (or more) elements are combined together.

#### *Regulating stability*

The former case is conventional; circRNAs facilitate RBPs to combine with mRNAs, thus stabilizing mRNAs and increasing translation (no example of destabilizing so far). CircNSUN2 combines with IGF2BP2 and high mobility group A (HMGA2) mRNA, forming a ternary complex that stabilizes mRNA. Then, HMGA2 upregulation induces epithelial-mesenchymal transition and promotes colorectal cancer aggressiveness [43]. Additionally, circPOK functions antithetically to its linear counterpart that encodes Pokemon, which suppresses tumors. CircPOK interacts with interleukin enhancer binding factor 2/3 (ILF2/3) complex and supports the stabilization of IL-6 and VEGF mRNA by ILF2/3. It also potentiates the occupancy of ILF2/3 on the IL-6 promoter. Collectively, circPOK regulates tumor cell secretome at both the transcriptional and posttranscriptional levels [122]. Similarly, the circFNDC3B-IGF2BP3-CD44 mRNA ternary complex stabilizes mRNA and upregulates CD44 [129].

#### *Regulating translation*

The latter case is more fascinating. One study described an unprecedented mode in which circRNA becomes stuck between mRNA and ribosome, acting like a brake and retarding translation. CircMALAT1 forms a ternary complex with paired box 5 (PAX5, a tumor suppressor) mRNA and ribosome through 11-complementary-bases and IRESs, respectively, leading to mRNA braking. It also sponges miR-6887-3p and activates the JAK2/STAT3 signaling. Both pathways promote HCC stem cells self-renewal [130]. Another study introduced a circRNA that interposes the translation initiation machinery. CircYap combines not only with its linear counterpart Yap mRNA, but also with eIF4G and PABP, which attach to the 5′-cap and 3′-tail, respectively. This tetramer obstructs the interaction between PABP and eIF4G, thus preventing Yap translation initiation [131]. Both studies depict how circRNA controls translation; however, one interrupts the elongation process, while another suppresses the assembly of initiation machinery. In contrast, circMYBL2 exacerbates acute myeloid leukemia by strengthening the interaction between PTBP1 and FMS-like tyrosine kinase 3 (FLT3) mRNA and promoting translation [132]. However, this study did not decipher how these molecules combine with each other (through binding domain, dynamic structure or sequence recognition?) and how this interaction improves translation efficiency.

Therefore, circRNAs exert biological roles at both the transcriptional/epigenetic and translational levels. Nevertheless, exact structures of the circRNA-protein-nucleic acid ternary complexes and precise processes of assembly, interconnection and possible disassembly have not been completely analyzed.

### Translocating or redistributing proteins

The most common intracellular translocation events occur between the nucleus and cytoplasm. In this section, according to the circRNA localization and protein redistribution guided by circRNAs, we placed the situations into four categories: (1) nuclear circRNAs cause the nuclear retention of proteins; (2) cytoplasmic or shuttling circRNAs facilitate the nuclear import of proteins; (3) cytoplasmic circRNAs cause the cytoplasmic retention of proteins; and (4) nuclear or shuttling circRNAs facilitate the nuclear export of proteins. The former two lead to nuclear translocation, while the latter two result in the cytoplasmic translocation of proteins.

#### *Increasing nuclear distribution*

CircAmotl1 promotes BC proliferation and invasion, and inhibits apoptosis. Mechanistically, nuclear circAmotl1 directly binds to c-myc, increasing its nuclear retention and stability, and then enhances the affinity of c-myc to downstream gene promoters [46]. Another study showed that circAmotl1 interacts with STAT3 and facilitates its nuclear translocation. Nuclear STAT3 binds to the DNMT3a promoter and activates transcription. DNMT3a can methylate the miR-17 promoter, thus upregulating miR-17-5p targets, including fibronectin, DNMT3a and STAT3. These factors form a positive feedback loop and promote fibroblasts proliferation, survival, adhesion, and migration, which together accelerate wound repair [133].

Similarly, circDNMT1 overexpression inhibits cellular senescence, which is mediated by stimulating autophagy. CircDNMT1 promotes the nuclear translocation of p53 and heterogeneous nuclear ribonucleoprotein D (hnRNPD, AUF1), inducing autophagy (by elevating LC3B level) and increasing DNMT1 translation (by stabilizing DNMT1 mRNA), respectively. Then, DNMT1 inhibits p53 transcription and decreases its total and cytoplasmic abundance while increasing its nuclear abundance [134]. Previous studies reported a dual role of p53 in controlling autophagy (cytoplasmic p53 represses autophagy, while nuclear p53 enhances autophagy) [165]. In turn, autophagy increases circDNMT1 levels, thus forming a complicated positive feedback network that suppresses senescence and promotes tumor progression [134]. Furthermore, circABCC1 [135] and circSOX4 [136] both increased the nuclear translocation of β-catenin and expedited tumor progression via the Wnt pathway.

#### *Increasing cytoplasmic distribution*

CircFoxo3 overexpression induces cardiac senescence and aggravates doxorubicin-induced cardiomyopathy by retaining the anti-senescence proteins inhibitor of DNA binding 1 (ID-1) and E2F1, as well as the anti-stress proteins tyrosine kinase 2 (FAK) and HIF1α in the cytoplasm, disrupting the functions of these TFs, which mainly work in the nucleus (FAK in the mitochondria) [137]. Additionally, circFOXP1 promotes the Warburg effect and gallbladder progression by increasing the cytoplasmic translocation of PTBP1 that binds to the 3′-UTR and coding region of pyruvate kinase L/R (PKLR) mRNA, protecting it from decay [138]. Similarly, circSTAG1 ameliorated astrocyte dysfunction and depressive-like behaviors induced by chronic unpredictable stress by capturing ALKBH5 in the cytoplasm and increasing m6A levels of fatty acid amide hydrolase (FAAH) mRNA [139, 166]. CircBACH1 [140] and circZFP609 [141] also fall in this situation.

A recent study showed that circCCAC1 promotes cholangiocarcinoma (CCA) tumorigenesis by sponging miR-514a-5p that targets YY1. Additionally, it can be packed into EVs and transferred to endothelial monolayer cells, resulting in disrupted barrier integrity and enhanced angiogenesis. Mechanistically, circCCAC1 retains EZH2 in the cytoplasm, demethylating the SH3 domain containing the GRB2-like 2 (SH3GL2) promoter. Subsequently, intercellular junction proteins are decreased, and cell permeability is increased. However, this effect is absent in CCA cells. Therefore, circCCAC1 plays dual roles in CCA cells and endothelial cells by sponging miR and redistributing protein, respectively [24].

#### *Other intracellular regions*

Three studies illustrated that circRNAs transport proteins to the nucleolus, membrane and mitochondria, respectively. CircERBB2 promotes gallbladder cancer proliferation and mainly accumulates in the nucleolus. Ribosome synthesis is crucial for malignancy, and cancer is characterized by abnormal ribosomal DNA (rDNA) transcription by Pol I [167]. CircERBB2 increases the nucleolar localization of proliferation-associated 2G4 (PA2G4), promoting PA2G4-TIFIA (Pol I transcription factor) interaction and thereby recruiting Pol I to the rDNA promoter [168]. However, a more detailed mechanism of how circERBB2 moves PA2G4 and whether circERBB2 anchors other proteins involved in rDNA transcription remains unknown [142]. CircSKA3 enhances BC invasion and metastasis by recruiting Tks5 to the membrane. This induces the formation of invadopodia where circSKA3 co-localizes with actin, Tks5 and integrin β1. Tks5 does not directly bind to integrin β1, but they precipitate each other, which indicates that a ternary complex bridged by circSKA3 forms [143].

A recent study found that mitochondria-encoded circRNAs (mecciRNAs) mecciND1 and mecciND5 can serve as molecular chaperones for replication protein A2 (RPA32) and hnRNPA1, respectively, and facilitate their entry into mitochondria by interacting with translocase of outer mitochondrial membrane 40 (TOM40). However, this effect in the posttranslational system is much weaker than that in the co-translational system, which suggests that mecciRNAs may only help newly synthesized peptides adopt structures that favor mitochondrial importation but have little impact on mature proteins [52]. MecciRNAs are distributed both inside and outside the mitochondria and may shuttle dynamically. Polynucleotide phosphorylase (PNPASE, a major mitochondrial RNA importation factor) interacts with most mecciRNAs and controls their mitochondrial levels [52].

In conclusion, protein redistribution is usually accompanied by enhanced or dampened activity and enabled or hampered access to targets, thus leading to boosted or arrested functions and corresponding downstream variations. However, the relationship between the transportation of proteins and circRNAs themselves has scarcely been studied.

### Other manners and summary

Several other studies also verified circRNA-protein interactions. However, because detailed mechanistic research is lacking, we are unable to classify them into any manners (Table 1) [144–160]. Of note, the techniques of identifying and characterizing the dynamic circRNA-protein interactions have been systematically reviewed, which mainly include RBP immunoprecipitation (RIP), RNA pull-down, RNase protection assay (RPA), crosslinking immunoprecipitation (CLIP), electrophoretic mobility shift assay (EMSA), fluorescence in situ hybridization (FISH) and immunofluorescence. RIP followed by RNA-sequencing/polymerase chain reaction profiles/verifies circRNAs that bind to specific proteins, while RNA pull-down followed by mass spectrometry/western blotting identifies/verifies proteins that bind to specific circRNAs. RPA and CLIP can map the sites of interactions, while EMSA can verify the formation of circRNA-protein complexes. FISH and immunofluorescence can detect the co-localization of circRNAs and proteins [8, 87–89].

Some circRNAs, such as circFOXO3 and circAmotl1, may implement diverse but similar functions by interacting with distinct proteins under different or dynamic circumstances. Some, such as circCcnb1 and circCCAC1, can even exert dual roles in different cell types. The underlying reason for this remains unknown and may be explained by external stimuli (specific microenvironmental or instructive factors) or internal features (dynamic tertiary structures or physicochemical properties). Some, such as circSMARCA5 and circPABPN1, may interact with the same protein that possesses various targets and thus be multifunctional.

It is necessary to state that five manners are not strictly exclusive, and occasional overlap appears among them. We distinguished them depending on the predominate effects observed and emphasized by researchers. Apart from the abovementioned ubiquity of ternary complexes, protein retention by circRNA is a sort of sequestration in some ways. Sometimes the formation of complexes occurs after the transportation of circRNPs, while sometimes alterations in protein activities or modifications lead to redistribution. Another aspect worth considering is the correlation between circRNA function and that of its parental gene. Nearly 50% circRNAs that interact with proteins act as positive regulators of parental genes, while approximately 20% act as negative regulators. The rest are unrelated or unknown yet. This phenomenon seems to not be subject to any specific rules. Some circRNAs directly interact with or indirectly regulate their cognate genes, linear counterpart transcripts or host gene products (DNA, mRNA and protein, respectively). However, most of them tread totally different pathways that lead to similar, opposite or unrelated outcomes compared to parental genes.

Interacting with the chief executors of life processes endows circRNAs with multiple biological functions. Among them, approximately 80% correlate with tumor progression or repression. Specifically, circRNAs engage in the overlapping modulation of tumorigenesis (proliferation and apoptosis), metastasis (invasion and migration), cell cycle, angiogenesis, metabolic reprogramming, senescence, autophagy, stemness, chemosensitivity, etc. The rest mainly include cardiovascular and neurological functions, inflammation and autoimmunity, wound repair and regeneration, myogenesis and adipogenesis, and undefined downstream effects. Though these studies involve extensive areas, they do not obey any other specific rules thus far, which indicates a large gap between basic research and clinical practice and reminds us that further explorations are indispensable.

## Conclusions and perspectives

In this review, we briefly summarize recent progress in circRNA metabolism and functions. Among these, circRNA-protein interactions hold climbing appeal to scientists and still lack a comprehensive research framework. Therefore, we collected almost all relevant studies to date and classified them into five patterns according to the direct effects that circRNAs exert on proteins: (1) altering interactions between proteins; (2) tethering or sequestering proteins; (3) recruiting proteins to chromatin; (4) forming circRNA-protein-mRNA ternary complexes; and (5) translocating or redistributing proteins. 

Despite fruitful progress, challenges and difficulties posed by theoretical deficiencies and technological restrictions exist. For example, what factors determine whether circRNAs act as scaffolds or decoys to support or impede proteins? Are there transcription repressors, other cis-elements (enhancers), chromatin modifiers or remodelers that are involved in circRNA regulation upon transcription? What upstream intrinsic properties or extrinsic factors elicit the formation of complexes from several specific molecules? Are there ncRNAs or other molecules (e.g., metabolites and ionic compounds) that combine with circRNPs? Are circRNAs involved in protein translocation to other organelles (e.g., the endoplasmic reticulum, Golgi apparatus and lysosome), subcellular compartments (e.g., euchromatic or heterochromatic territories, the nuclear lamina and membrane-less organelles formed by phase separation) or extracellular spaces (e.g., the matrix and adjacent or distant cells)? Do circRNAs affect the translocation of other molecular species? How can we profile circRNAs located in other organelles or deliver circRNAs to specific subcellular compartments? How can we analyze the exact structure of circRNPs beyond the binding sites to interpret the conformational and functional changes of proteins? How can we improve the canonical techniques (e.g. RIP, RNA pull-down, RNA-sequencing) in view of the low abundance and special structure of circRNAs? How can we capture the real-timecircRNA-protein interactions in vivo and display the dynamic processes? How can we design the animal models and conduct pre-clinical studies targeting circRNPs?

At present, the study on circRNA-protein interaction is still staying at the very beginning stage and far away from translational medicine. Though their unique structures and resistance to RNA decay machinery enable them to act as ideal diagnostic biomarkers and therapeutic targets, regrettably, not a single circRNA-based medical application has been approved so far. However, some excellent studies that we have mentioned above have shown promising results and achievements. For example, in the study of circRNA SCAR, a mitochondria-targeting nanoparticle (mito-NP) platform that specifically delivers encapsulated vectors to the mitochondria was designed and this enables mitochondria-targeted therapy to tackle metaflammation associated diseases [50]. In the study of circPan3, cia-cGAS and circKcnt2, the researchers generated circRNA-knockout mice by targeting the ICSs in flanking introns of circRNAs with CRISPR/Cas9 technology, which avoids affecting corresponding parental genes [78, 106, 128]. This provides convenience for basic research and possibilities for translational practice. CircCcnb1 has totally inverse roles in different cells, which makes it a potential therapeutic agent for p53 mutant tumors because it causes less damage to adjacent benign p53 wild-type tissues [100]. Proteins control the biological activities in all aspects. CircRNA-protein interactions make changes to proteins and thus make a difference in life processes, which sparks our interest in devising novel clinical strategies that manipulate (interfere or utilize) circRNAs based on circRNA-protein interaction.

## Data Availability

Not applicable.

## Abbreviations

circRNA: circular RNA
miR: microRNA
mRNA: messenger RNA
CDR1as: Antisense transcript of cerebellar degeneration-related protein 1
ceRNA: competing endogenous RNA
Pol II: RNA polymerase II
ICS: intronic complementary sequence
RBP: RNA binding protein
m6A: N6-methyladenosine
METTL: Methyltransferase-like
YTHDC: YTH domain-containing
YTHDF: YTH domain family
EV: extracellular vesicle
HCC: hepatocellular carcinoma
Ago2: Argonaute2
eIF4G: eukaryotic translation initiation factor 4 gamma
ncRNA: non-coding RNA
lncRNA: long non-coding RNA
IGF2BP: insulin like growth factor 2 mRNA binding protein
IL: interleukin
IRES: internal ribosome entry site
UTR: untranslated region
ORF: open reading frame
circRNP: circRNA-protein complex
MDM2: mouse double minute 2
FOXO3: forkhead box O3
CDK: cyclin-dependent kinase
YBX1: Y-box binding protein 1
Ccn: cyclin
GSK3β: glycogen synthase kinase 3 beta
VEGF: vascular endothelial growth factor
ACC1: acetyl-CoA carboxylase 1
AMPK: AMP-activated protein kinase
TF: transcription factor
GC: gastric cancer
HuR: human antigen R
cGAS: cyclic GMP-AMP synthase
SRSF: serine and arginine rich splicing factor
PTBP: polypyrimidine tract binding protein
PABP: poly-adenosine binding protein
BC: breast cancer
DNMT: DNA methyltransferase
ILF: interleukin enhancer binding factor
Yap: Yes1-associated transcriptional regulator
STAT3: signal transducer and activator of transcription 3
LC3B: light chain 3 beta
Pol I: RNA polymerase I
mecciRNAs: mitochondria-encoded circRNAs
hnRNP: heterogeneous nuclear ribonucleoprotein
